# Mutation Profiling of the Hepatitis B Virus Strains Circulating in North Indian Population

**DOI:** 10.1371/journal.pone.0091150

**Published:** 2014-03-17

**Authors:** Amit Tuteja, Abu Baker Siddiqui, Kaushal Madan, Rohit Goyal, Shalimar, Vishnubhatla Sreenivas, Navkiran Kaur, Subrat K. Panda, Krishnamoorthy Narayanasamy, Swati Subodh, Subrat K. Acharya

**Affiliations:** 1 Institute of Molecular Medicine, New Delhi, India; 2 Amity Institute of Biotechnology, Amity University, Noida, India; 3 Open Source Drug Discovery Unit, Council of Scientific & Industrial Research, New Delhi, India; 4 Department of Gastroenterology, All India Institute of Medical Sciences, New Delhi, India; 5 Department of Pathology, All India Institute of Medical Sciences, New Delhi, India; 6 Department of Statistics, All India Institute of Medical Sciences, New Delhi, India; CRCL-INSERM, France

## Abstract

**Aims:**

The aim of this study was to investigate the genomic mutations in the circulating Hepatitis B virus strains causing infection in the Indian population. Further, we wanted to analyze the biological significance of these mutations in HBV mediated disease.

**Methods:**

222 HBsAg positive patients were enrolled in the study. The genotype and mutation profile was determined for the infecting HBV isolate by sequencing overlapping fragments. These sequences were analyzed by using different tools and compared with previously available HBV sequence information. Mutation Frequency Index (MFI) for the Genes and Diagnosis group was also calculated.

**Results:**

HBV Genotype D was found in 55% (n = 121) of the patient group and genotype A was found in 30% (n = 66) of samples. The majority (52%) of the HBV-infected individuals in the present study were HBeAg-negative in all the age groups studied. Spontaneous drug associated mutations implicated in resistance to antiviral therapy were also identified in about quarter of our patients, which is of therapeutic concern. The MFI approach used in the study indicated that Core peptide was the most conserved region in both genotypes and Surface peptide had highest mutation frequency. Few mutations in X gene (T36A and G50R) showed high frequency of association with HCC. A rare recombinant strain of HBV genotype A and D was also identified in the patient group.

**Conclusions:**

HBV genotype D was found out to be most prevalent. More than half of the patients studied had HBeAg negative disease. Core region was found to be most conserved. Drug Associated mutations were detected in 22% of the patient group and T36A and G50R mutations in X gene were found to be associated with HCC.

## Introduction

Hepatitis B virus (HBV) is the most common cause of chronic hepatitis, Cirrhosis and Hepatocellular Carcinoma (HCC) globally [1], [2]. HBV is a DNA Virus with 3200 nucleotides and has four Open Reading Frames (ORFs) encoding for hepatitis B surface or envelope proteins, core peptide, X peptide and DNA-polymerase enzyme. It replicates through RNA intermediate and therefore uses a reverse transcriptase to form a c DNA. The reverse transcriptase being a poor proof reader is known to introduce synonymous (silent) as well non-synonymous substitutions in the HBV genome. Depending upon the genetic heterogeneity of HBV(>8%) 8 genotypes A through H has been identified [3],[4],[5],[6],[7],[8],[9],[10],[11]. More recently, two additional genotypes (I and J) were tentatively proposed [12], [13], [14]. The genotypes and the non-synonymous mutations has been reported to be clinically relevant, particularly in the spontaneous HBeAg clearance, transmission potential, disease progression, hepatocarcinogenesis, and response to therapy [1], [15], [16].

In India, predominantly prevalence of HBV Genotype A and D have been reported [1], [17], [18], [19], [20], [21], [22]. However, reports on spontaneous HBV mutation in these particular genotypes in Indian patients with chronic HBV infection at various stages are not available. Indians are a distinct ethnic group and may have distinct virus-host interaction with resultant effect on the virus. Further, information on the spontaneous base line mutation status in these patients may influence therapeutic decisions. Use of nucleos(t)ides is a common practice among physicians in India. It is possible that many patients may have drug resistant mutants to a particular oral anti-viral and if identified before therapy would be relevant in the management of such patients.

The present study was designed to identify the mutation profile by whole genome sequencing in HBV, isolated from chronically infected HBV patients in North-India. The study also aims to identify differences of mutation among prevalent genotypes and their clinical relevance.

## Materials and Methods

### 1. Ethics Statement

Ethical clearance from institutional review board of All India Institute of Medical Sciences was obtained for this study. Also informed written consent was obtained from each patient to participate in the study in presence of a witness and the consulting doctor. The written consent was obtained in English or Hindi (vernacular language) as the case may be.

### 2. Patient's Information

Two hundred twenty two consecutive patients with chronic HBV infection and attending the liver clinic at the department of gastroenterology, All India Institute of Medical Sciences, New Delhi, India, during January 2007 to March 2009 were included in the study. Diagnosis of chronic HBV infection was made by documenting HBsAg positivity at 6 month interval in each (N = 217), or by persistence presence of Anti-HBc with detectable HBV DNA in sera over 6 months duration (N = 5).

Diagnosis of asymptomatic healthy carrier (AC) (N = 40), Chronic hepatitis (CHB) (N = 152), Cirrhosis (LC) (N = 20) and hepato-cellular carcinoma (HCC) (N = 10) was made by conventional and accepted criteria [23] which included, clinical, biochemical, endoscopic, radiological and histological characteristics for each of these liver diseases. Those with HIV and HCV co-infection and with history of alcohol consumption of >20 gm/day were excluded from the study.

### 3. Sample Processing

#### 3.1 Serological Detection

Sera was collected from each patient, before any therapeutic intervention and was stored at −80°C. Each patient was tested for liver function test [Serum bilirubin, AST (Aspartate Aminotransferase), ALT (Alkaline Phosphatase), Total protein and Serum albumin]. Patients were also tested for hepatitis B surface antigen (HBsAg), hepatitis B early antigen (HBeAg) and hepatitis C virus (HCV) using commercial ELISA kits (Biorad Laboratories, California, USA).

#### 3.2 Viral DNA Isolation

HBV DNA was extracted from 200 µl serum using the High Pure Viral Nucleic Acid Kit (Roche Diagnostics, Germany) according to the manufacturer's instructions. HBV DNA was eluted in 40 µl and was stored at −20°C until further use.

#### 3.3 HBV DNA Quantitation

HBV DNA was quantified by SYBR Green based real-time detection PCR assay using the Light Cycler 480 Real Time System (Roche, Germany). HBV quantification was done using a previously reported method with minor modifications [24]. In Brief, serially diluted WHO HBV control of 1×10^6^ IU/ml (NIBSC, UK) was taken as reference to calibrate an internal laboratory control. The dynamic range of HBV detection was between 10^2^ to 10^8^ copies/ml. 143 bp region of the surface gene (region 303–446 bp) was amplified with Forward primer (HBV-FP) (5′ACTCACCAACCTCTTGTCCT3′) and Reverse primer (HBV-RP) (5′GACAAACGGGCAACATACCT3′). The thermal cycling conditions were, initial denaturation at 95°C for 10 minutes; 35 cycles of (denaturation at 95°C for 15 seconds, annealing at 60°C for 30 seconds and extension with data acquisition at 72°C for 30 seconds). Appropriate positive and negative controls were set up with the clinical samples to be analyzed.

#### 3.4 PCR Amplification

Full genome amplification was performed by overlapping genome fragment amplification as previously described [25]. The following thermal cycling parameters were used for 35 cycles of PCR: denaturation at 94°C for 40 seconds, annealing at 56°C for 1 min and elongation at 73°C for 2.5 mins. Where the primary PCR amplification was negative, a nested PCR was carried out to amplify the desired region. Thermal cycling parameters used for nested PCR were same as that for the outer PCR. All PCR reactions were carried out using the Go Taq® Flexi DNA Polymerase (Promega Corp, USA). The PCR products were visualized on 1% agarose gel stained with ethidium bromide and purified using AMPure® kit (Agencourt Bioscience Corporation, USA).

#### 3.5 Sequencing

Nucleotide sequencing of the PCR fragments was performed with the BigDye® Terminator v3.1 Cycle Sequencing kit (Applied Biosystems, USA), with appropriate primers, and sequenced using the 3730 DNA sequencer (Applied Biosystems, USA). Base Calling was done using Sequencing Analysis Software v5.3.1 with KB™ Basecaller v1.4 (Applied Biosystems, USA). All sequences were edited manually. The sequences obtained were submitted to Genbank database (http://www.ncbi.nlm.nih.gov/genbank/). Genbank Accession numbers (JY084622 - JY085088).

### 4. Strain Characterization

#### HBV Genotypes and Mutation identification

A reference sequence based HBV genome assembly was done for the individual fragments of the HBV isolates by aligning with NCBI reference sequences [Genotype A (Accession no. AF090842), Genotype D (Accession no. M32138)] using SeqScape® Software version 2.5 (Applied Biosystems, USA). Previously published whole genome sequences of HBV (A- 23 sequences and D-22 sequences) from Indian subcontinent were also taken for comparative analysis. The genotypes were determined based on the identity in the S or preS gene using Oxford HBV Automated Subtyping Tool version 1.0 [26], [27]. The mutation analysis of sequences was done using SeqScape® Software version 2.5 (Applied Biosystems, USA), Variant Reporter version 1.1 (Applied Biosystems, USA) and HBV- Resistance interpretation tool algorithm version 03-2007 [28] and Geno2Pheno(HBV) tool [29].

### 5. Statistical analysis

χ^2^ test and Fisher's exact test were used for statistical analysis and were denoted as mean ±1 standard deviation. *P* value of less than 0.01 was considered significant.

### 6. Mutation Frequency Index (MFI)

MFI was calculated for all four HBV peptides (Surface, polymerase, X and Core). Total number of amino acid mutations was calculated individually for these peptides in all the samples within a diagnostic group. The Quality score cut-off for sequence data in the region of mutations was kept at Phred score, Q≥20 [30]. The MFI was then calculated using the following formula
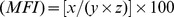



MFI – Mutation Frequency Index

x - Total Number of Mutations observed in amino acid sequences

y - Total Number of samples in a group

z – Length of amino acid sequence

The total number of independent mutations observed in all the respective peptides was plotted by distributing the peptide length into bin size of 30 amino acids in order to get an overview of the regions highly prone to mutations within the peptide. Heat maps were generated for the individual peptide datasets to graphically depict the distribution of mutation frequency across the peptide. Briefly the number of mutations per 30 peptide bases were plotted on x axis and regions across were color coded with red depicting highest mutation frequency and green least mutation frequency.

## Results

### 1. Patient's Profile

The basic demographic, virological and biochemical properties from 222 patients have been depicted in Table 1. 95 patients were found out to be HBeAg positive (43%) and 115 were HBeAg negative (52%). Fifty five percent (n = 121) and thirty percent (n = 66) patients had Genotype D and A HBV isolates respectively. In 7 patients mixed isolates of both genotypes A and D HBV could be identified. One patient had a HBV DNA isolate with recombinant genotype A and D. In this isolate a portion of polymerase gene and X gene of genotype D was inserted in the backbone of genotype A, details of which has been described later. In the remaining twelve percent (27/222) cases the genotype could not be determined. The distribution of HBV genotypes did not vary significantly among AC, CHB, LC and HCC groups (Table 1). Overall we observed a greater ratio of male patients across all diagnostic groups.

**Table 1:** Information summary of the patients participating in the study.

	AC (n = 40)	CHB (n = 152)	LC (n = 20)	HCC (n = 10)
Age (years)^b^	31.46±10.28	30.2±10.6	38.25±16.19	42.9±16.35
Gender (M/F)	30/10	133/18	19/1	9/1
ALT (IU/L)^b^	36.96±17.46	98.85±94	77.15±85.29	90.2±82.3
AST (IU/L)^b^	39.12±16.6	70.06±62.67	117.4±171.05	151.2±153.64
HBeAg(+/−)	9/31	75/67	6/12	5/5
HBV DNA (log copies/mL)^b^	3.07±0.35	4.58±1.64	3.90±1.73	3.83±2.5
HBV/A^a^	17 (42.5)	40 (26.4)	5 (25)	4 (40)
HBV/D^a^	20 (50)	87 (57.6)	10 (50)	4(40)
HBV/Genotype Unclassified	1 (2.5)	19 (12.5)	5 (25)	2 (20)
Mixed	1(2.5)	6(4)	-	-
Recombinant	1(2.5)	-	-	-

a Data shown as number and (percent of total).

b Data shown as mean value ±1 S.D.(Standard Deviation).

### 2. Mutation Frequency Index (MFI) of the Infecting HBV isolate

**Figure 1:**
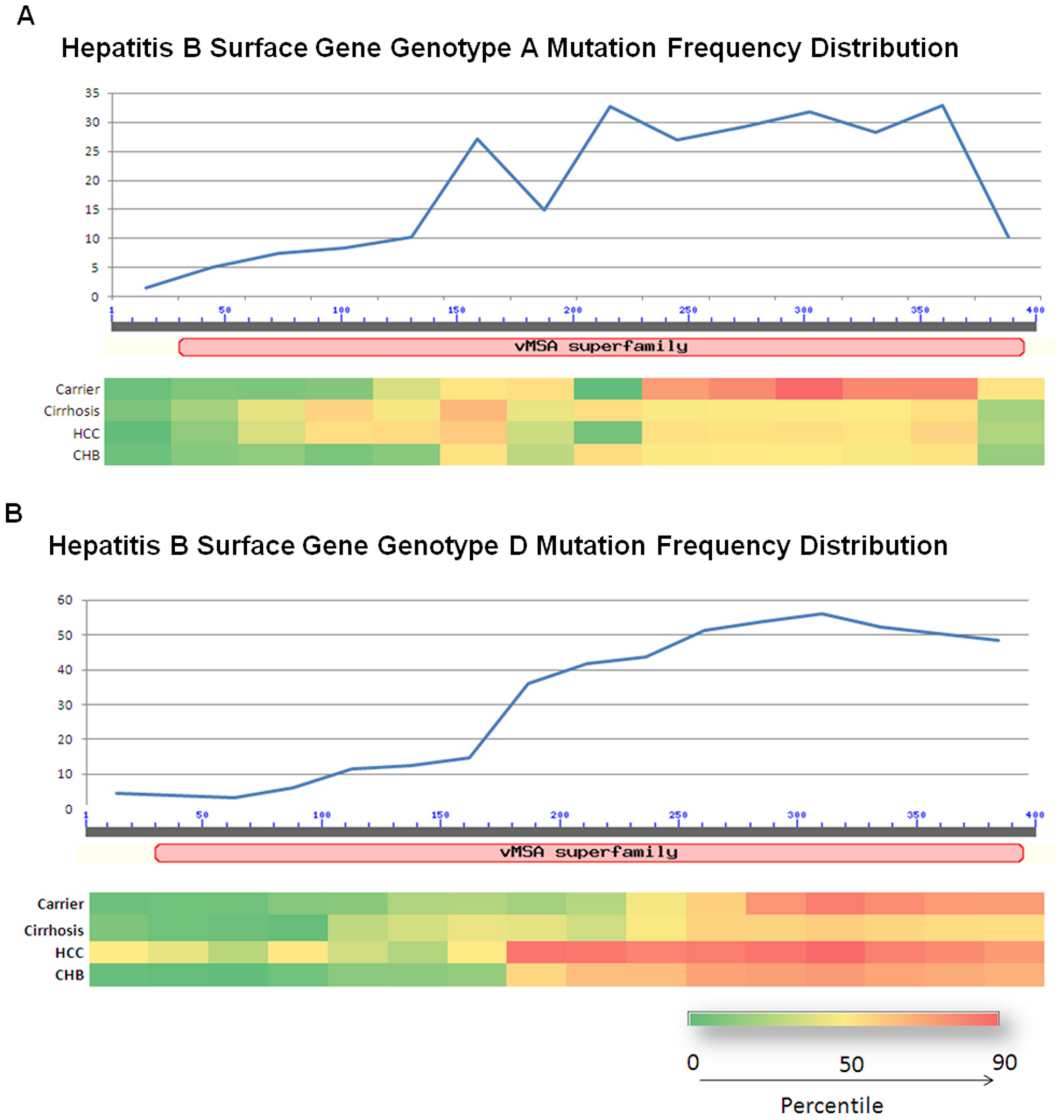
The distribution of Mutation Frequency in hepatitis B virus surface gene of A Genotype A and B Genotype D.

**Figure 2:**
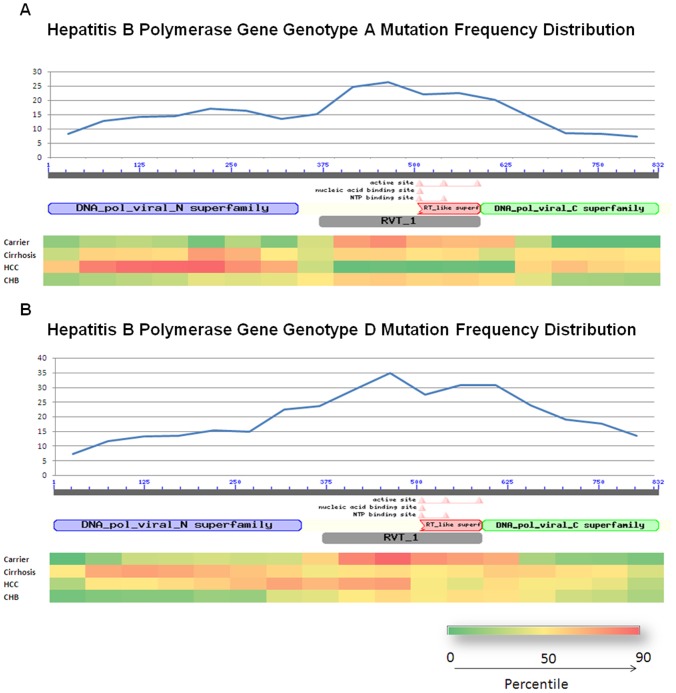
The distribution of Mutation Frequency in hepatitis B virus polymerase gene of A Genotype A and B Genotype D.

Mutation Frequency Index (MFI) distribution among different diagnosis groups is summarized in Table 2. Overall an increase in MFI was observed in LC and HCC cases as compared to CHB cases. Within most diagnosis groups the MFI was higher for HBV genotype D compared to genotype A. The observed average MFI was least for core (7.4) followed by X (9.3), P (21.7) and S (26.2) gene (Table 2). Heat maps were generated for the Surface peptide (Figure 1) and Polymerase peptide (Figure 2) to graphically depict the distribution of mutation frequency.

**Table 2:** MFI distribution among different HBV diagnostic groups, genotypes and peptides.

	Diagnosis								
	AC		CHB		LC		HCC		
MFI (Diagnosis)	No data^a^		19.6		21		22.6		
	HBV Genotype A	HBV Genotype D	HBV Genotype A	HBV Genotype D	HBV Genotype A	HBV Genotype D	HBV Genotype A	HBV Genotype D	Avergae MFI (Peptide)
MFI (Genotype)	NC	NC	13.5	23.5	16.4	24.2	21	24.3	-
Surface Gene	36.9	28.9	14.5	31.6	20.6	25.2	18.9	32.5	26.2
Polymerase Gene	17.4	26.7	15.4	17.9	19.6	31.1	24.1	22	21.7
X Gene	NA	NA	8.6	8.8	8.6	6.2	18.2	5.1	9.3
Core Gene	NA	NA	8	9.7	7.2	6.5	8.7	4.3	7.4

a Sufficient data was not available for Asymptomatic carriers (AC) for Core and X gene.

### 3. Mutations in Hepatitis B Virus genome

Overall 51 independent amino acid substitutions were observed in all genes. Of which 6 significant substitutions were observed in surface peptide of HBV genotypes A & D (Table 3). The distribution of these substitutions was significantly different with respect to HBV genotypes. Interesting to note that T127P surface peptide mutation was documented in 54.5% of the Genotype D isolates in comparison to 6.1% of Genotype A. This mutation is in the region of immunogenic epitope of ‘a’ determinant of HBsAg which binds to anti-HBs antibody. Therefore such isolates if infects another individual may result in vaccine escape mutants. Similarly, 22 amino acid substitutions were observed in reverse transcriptase region of polymerase gene. Of these 15 mutations were associated with HBV genotype A and 7 with genotype D (Table 4). In HBx peptide of HBV, 11 amino acid substitutions were observed in all, of which 5 mutations were associated with HBV genotype A and 6 with genotype D (Table 5). 11 amino acid substitutions were observed in Core peptide for both HBV genotype A & D (Table 6). Of these 2 were associated with HBV genotype D and 9 were associated with genotype A.

**Table 3:** Distribution of Mutations in Surface gene.

		Genotype A (n = 58)		Genotype D (n = 105)		
S.no	Amino acid substitution	Frequency	Percentage	Frequency	Percentage	P Value by Fisher's Exact test
1.	F8L	7	10.6	2	1.7	<0.05
2.	T118V	3	4.5	17	14	<0.05
3.	T127P	4	6.1	66	54.5	<0.05
4.	S207N	24	36.4	12	9.9	<0.05
5.	L209V	7	10.6	2	1.7	<0.05
6.	S210M	6	9.1	2	1.7	<0.05

**Table 4:** Distribution of Mutations in Reverse Transcriptase region of Polymerase peptide.

		Genotype A (n = 66)		Genotype D (n = 121)		
S.no.	Amino acid substitution	Frequency	Percentage	Frequency	Percentage	P Value by Fisher's exact test
1.	D7A	21	31.8	9	7.4	<0.05
2.	H9Y	15	22.7		0	<0.05
3.	A21S		0	17	14	<0.05
4.	Y54H	3	4.5	19	15.7	<0.05
5.	F122L	6	9.1	29	24	<0.05
6.	M129L	22	33.3	18	14.9	<0.05
7.	Y135S	4	6.1	67	55.4	<0.05
8.	V163I	21	31.8	13	10.7	<0.05
9.	A223S	11	16.7	7	5.8	<0.05
10.	N248H	8	12.1	59	48.8	<0.05
11.	I253V	24	36.4	14	11.6	<0.05
12.	D263E	5	7.6	32	26.4	<0.05
13.	Y305A	12	18.2	10	8.3	<0.05
14.	L308A	11	16.7	5	4.1	<0.05
15.	A329P	10	15.2	7	5.8	<0.05
16.	Y335N	11	16.7	9	7.4	<0.05
17.	L336M	6	9.1	29	24	<0.05
18.	N337F	11	16.7	9	7.4	<0.05
19.	Y339T	12	18.2	9	7.4	<0.05
20.	V341S	11	16.7	9	7.4	<0.05
21.	A342C	11	16.7	9	7.4	<0.05
22.	Q344P	11	16.7	8	6.6	<0.05

**Table 5:** Distribution of Mutations in X gene.

		Genotype A (40)		Genotype D (74)		
S.no.	Amino Acid Substitution	Frequency	Percentage	Frequency	Percentage	P Value by Fisher's exact test
1.	S11P	33	50	2	2.7	<0.05
2.	A31S	31	47	1	1.4	<0.05
3.	R32G	31	47	1	1.4	<0.05
4.	S33P	1	1.5	13	17.6	<0.05
5.	V37L	37	56.1	3	4.1	<0.05
6.	Q87H	4	6.1	21	28.4	<0.05
7.	L98C	14	21.2	5	6.8	<0.05
8.	E126R	1	1.5	22	29.7	<0.05
9.	V133Y	5	7.6	21	28.4	<0.05
10.	A144H	1	1.5	21	28.4	<0.05
11.	P145Q	2	3	24	32.4	<0.05

**Table 6:** Distribution of Mutations in Core gene.

S.no	Amino Acid Substitutions	Genotype A (34)		Genotype D (82)		P Value by Fisher's exact test
		Frequency	Percentage	Frequency	Percentage	
1	M30L	24	70.6	1	1.2	<0.05
2	D31A	24	70.6	1	1.2	<0.05
3	P34H	24	70.6	1	1.2	<0.05
4	C77G	5	14.7	28	34.1	<0.05
5	T120N	10	29.4	3	3.7	<0.05
6	T138Q	13	38.2	4	4.9	<0.05
7	S150V	20	58.8	2	2.4	<0.05
8	P159Q	30	88.2	1	1.2	<0.05
9	A160S	11	32.4	1	1.2	<0.05
10	Y161I	18	52.9	3	3.7	<0.05
11	Q211H	1	2.9	36	43.9	<0.05

Precore promoter mutation (G1896A) was also observed in 46% (56/121) of HBV Genotype D and 25% (17/66) of HBV Genotype A patients.

### 4. Drug Associated Mutations

Previously reported Drug associated mutations were also observed in our study group. The mutations were reported to be associated with Nucleos(t)ide analogues used for therapy in HBV infections. The distribution of drug associated mutations with respect to genotype and diagnosis is presented in Table 7. In all 74 mutations in 42 of the 187 isolates derived from treatment naïve patients, genotypic mutations known to cause resistance to licensed oral antiviral nucleos(t)ide, could be identified.

**Table 7:** Distribution of Drug Associated Mutations with HBV Diagnosis.

Amino acid Position in Reverse Transcriptase	Mutation	AC		CHB		LC		Total (187)	Mutation associated with Drug(s) resistance
		A	D	A	D	A	D		
80	L80*	4	1	8	2	2	4	21	Lamuvidine/Telbivudine
169	I169N	1						1	Entecavir
173	V173L			2	1			3	Lamuvidine
180	L180*			2	1		1	4	Lamuvidine/Entecavir
181	A181G			2	1		1	4	Lamuvidine/Adefovir
202	S202I				1			1	Entecavir
204	M204V			2			1	3	Lamuvidine/Entecavir/Telbivudine
215	Q215S		2	1	2			5	Adefovir
233	I233V		1	2	2		1	6	Adefovir
236	N236I		1	5	6		1	13	Adefovir
250	M250V		1	5	6		1	13	Entecavir

### 5. Novel Mutations

Many novel mutations were observed which showed a high degree of association with HBV genotype (Table 8).

**Table 8:** Novel Mutations identified in our study.

Amino acid substitutions	Genotype A			Genotype D			p Value	Gene
	Frequency	percentage	Total	Frequency	percentage	Total		
T49S	0	0	34	46	56.1	82	<0.01	Core
E64N	2	5.9	34	43	52.4	82	<0.01	Core
L30F	3	7.5	40	35	47.3	74	<0.01	X Gene
Q87H		0	40	14	18.9	74	<0.01	X Gene
M103D	11	27.5	40	3	4.1	74	<0.01	X Gene
D45L	0	0	66	69	57.0	121	<0.01	Polymerase
T70X	17	25.8	66	0	0	121	<0.01	Polymerase
S74X	4	6.1	66	0	0	121	<0.01	Polymerase
F19X	2	3.4	58	67	63.8	105	<0.01	Surface
V47G	17	29.3	58	4	3.8	105	<0.01	Surface

### 6. Recombinant Strain

**Figure 3:**
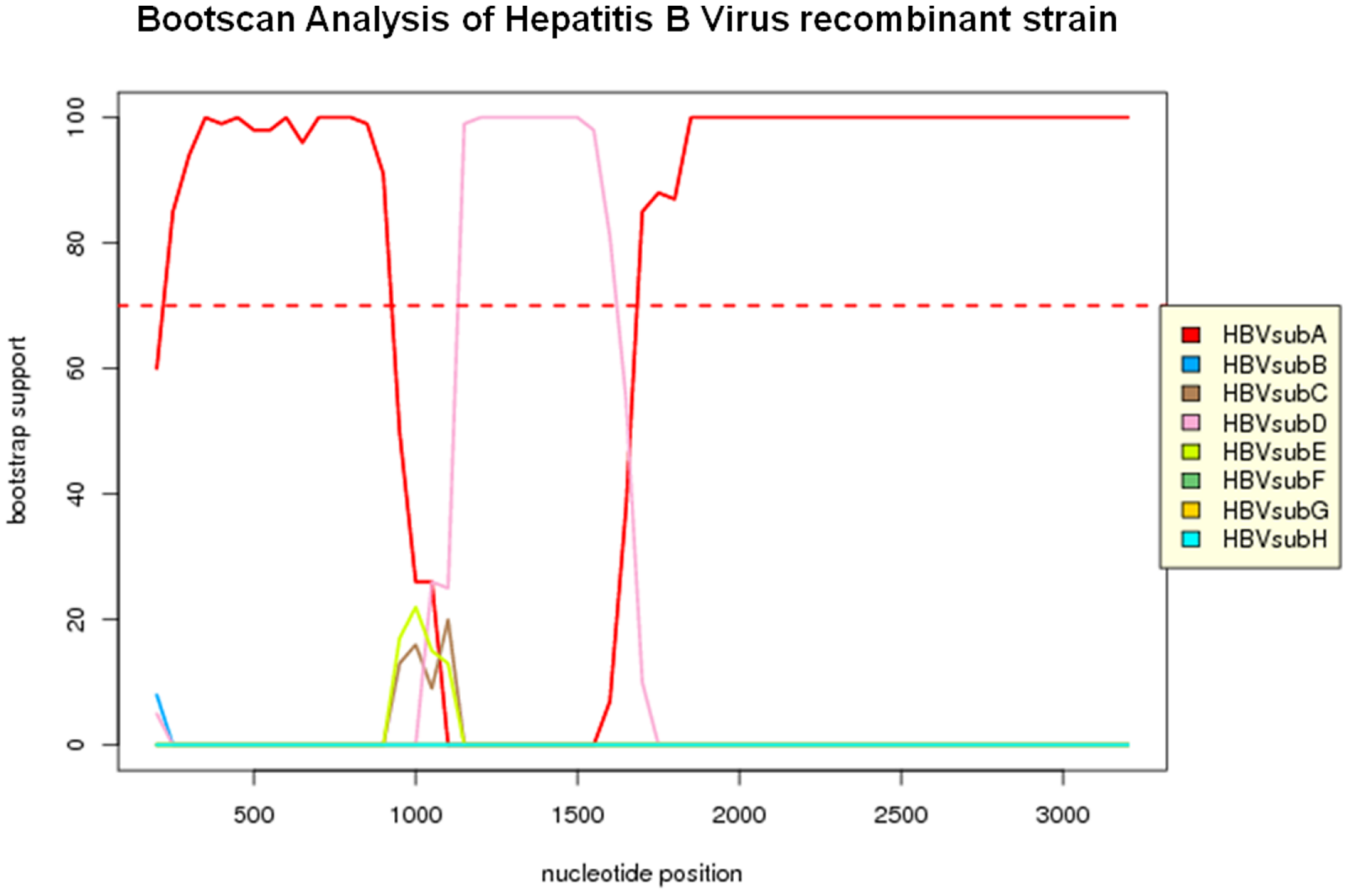
SIM plot showing the recombination analysis of the whole genome of HBV isolate TCGA5889 with respect to all the 8 known HBV genotypes. The bootscan analysis of the SIM plot was performed with a window size of 400 and a step size of 50.

Among the clinical samples collected and analyzed in the present study, a rare recombinant strain (TCGA 5889) of HBV genotype A and D was also identified. TCGA5889 had sequence between 986–1838 bp (852 bp) similar to genotype D, on a backbone of genotype A (Figure 3). Partial polymerase (986 bp–1623 bp) and complete X gene was similar to genotype D. The polymerase peptide had a stop codon at position 528 due to a T/A transversion thereby generating a truncated polymerase peptide (527aa). Core and surface peptides showed high similarity to genotype A. The recombinant and mixed genotypic strains were cloned and sequenced for confirmation.

## Discussion

India is a vast country, comprising of multiracial communities and geographic divide. It is therefore expected that infectious and chronic disease patterns may differ between various geographic regions [31].

In the present study HBV Genotype D (55%) and Genotype A (30%) were most frequent genotypes and similar findings have been reported earlier from India [19], [32], [33]. Most of the studies reported from India indicate that about 50–60% of HBV isolates are genotype D and about 30–40% are genotype A. However in the present study 10% of the samples could not be classified into genotypes due to lack of amplification of HBV DNA. The reason for this can be either absence or disruption of intact primer binding site in these samples or low HBV viral load or both.

Some reports indicate that globally HBeAg negative HBV infection is increasing [34], [35]. HBeAg negative chronic HBV infection with ongoing hepatic necroinflammation are known to cause more progressive disease, they are more difficult to treat and may have more frequent association with primary liver cancer. In the present study more than half of HBV isolates were HBeAg negative and often associated with Genotype D (G1896A) HBV infection (pre-core mutation). However, quarter of HBeAg negative isolates has genotype A (A1762T and G1764A) HBV infection (Basal Core Promoter mutation)

The PC/BCP mutations were also identified in (12/95) cases with HBeAg positive infection, however in these cases, the double mutation was often accompanied by a change at position 1753, from T to C or G. In addition, other point mutations upstream and downstream of A1762/G1764 at positions 1753, 1766 and 1768 have been described. [36], [37], [38]

An important aspect of this study was to understand the distribution and significance of HBV mutants associated with infecting isolates circulating in the North Indian population. Random mutation and natural selection drives the evolution and as a result, some genes, nucleotides, amino acids are conserved and others are not. This way organism can save resources to repair them and avoid lethal mutations. We have used following two approaches to understand the mutation profile of the HBV virus circulating in the patients under study.

- The first approach termed as Mutational Frequency Index (MFI) gives an overall estimate of the conserved and variable regions in the genome and
- The second approach deals with detailed analysis of the individual mutations in all four genes and their association with genotype and disease progression.

However the limitation of second approach would include that sequencing of PCR amplified product may not indicate the quasi species variation and relevant SNP identification.

Since HBV replication involves an error-prone reverse transcription step, the rate of nucleotide change during replication is higher than that found for other DNA viruses and is more similar to the rate observed for the slower-evolving RNA viruses [39]. The rate of HBV evolution in hepatitis B virus infected individuals has been estimated to be 1.5×10^−5^ to 5×10^−5^ nucleotide substitutions per site per year [40], [41], [42]. At a time an isolate may have many mutations (synonymous as well as non- synonymous). This frequency of mutation may have a pattern across genotypes, diagnosis groups and a population set. To study these frequencies across various situations as above, we used MFI. The relevant findings and importance of MFI is ascertained by following observations.

The distribution of MFI was analyzed across genotypes and disease groups to check if the distribution is random and how it can contribute in survival of HBV. Across genes we have observed that average MFI was lower for HBx and core peptides as compared to surface and polymerase peptides. These results are in concordance with previous studies, which have discussed high degree of conservation for Core and X genes [1], [43], [44]. The rate of mutation is relatively higher in Surface and polymerase gene which is possibly due to host immune pressure or due to wide scale use of nucleos(t)ide analogues to treat chronic HBV infection in general.

Also, the variability in polymerase gene was found to be highest in the middle (i.e. Reverse transcriptase region) as compared to C-terminal and N-terminal domains (Figure 2). This region is important as most of the antivirals (Nucleot(s)ide analogs) are targeted towards the reverse transcriptase region. The higher MFI in this region might lead to lower response by patients to antiviral treatments. This prediction need to be verified though, by large scale prospective studies.

In Diagnostic groups MFI was higher in LC and HCC groups as compared AC and CHB (Table 2). This may be due to accumulation of certain mutations (PC/BCP) in viral genome in patients with advanced liver disease and long history of infection. This has also been reported earlier [45], [46], [47].

Also MFI was observed to be higher for HBV genotype D as compared to HBV Genotype A, across all genes and diagnosis groups. This may be due to higher prevalence of Genotype D in our population group.

The MFI approach can have applications in case of clinical trials of nucleot(s)ide based antiviral therapies where it can help clinicians in getting an overview of the change in mutation frequency in responders v/s non responders of therapy and also in early prediction of drug resistance.

Mutations in ***surface peptide*** are clinically important in both HBV prevention (through vaccination) and diagnosis. Regarding their effects on immunization, large HBV vaccination programs in endemic regions have revealed a 2% to 3% incidence of vaccine escape mutants resulting from alterations in the HBsAg protein. The S gene of HBV has three open reading frames (ORF), including preS1, preS2 and S region. In the present study of the 27 mutations observed in this gene 7 were present in “α” determinant region (121–149 aa). All the 5 patients with Anti HBc positive (Occult Hepatitis) profile were found to contain mutations in this region (P127T). This is in concordance with results cited earlier [48], [49], [50]. The mutations in this region are of great public health significance because patients harboring HBV with these surface mutants do not exhibit quantifiable HBsAg, but remain infectious. HBV infection remains detectable by HBV-DNA and/or HBeAg testing [51].

The ***polymerase gene*** product is needed for encapsidation of viral RNA into core particles [52] and conversion of the pregenomic viral RNA molecule into genomic viral DNA. Although the HBV reverse transcriptase is highly conserved, infrequent mutations have been described [53]. The following mutations in RT region of polymerase gene A21S, Y54H, F122L, Y135S, were reported to be associated with HBV genotype D in previous studies [21], [25], [54], [55]. Similarly, several studies have reported association of D7A, M129L, V163I and I253V with HBV genotype A [21], [25], [56], [57].Both observations were in concordance with our results.

The ***HBV X gene*** has the smallest ORF, which encodes 155 amino acids. HBx has been reported to enhance transcription from the HBV genome and is also capable of up-regulating transcription from a wide variety of cellular and viral promoters. This function is mediated through protein-protein interaction involving cellular factors. Several X gene mutations have been reported to be associated with occurence of HCC [58]. Few of these mutations at a.a. position 127, 130 and 131 were extensively studied for their high prevalence rate in HCC cases [59], [60]. In our study we have observed K130M, T36A and G50R to be significantly associated with HCC (Table 9). K130M and V131I are translated due to basal core promoter mutations Adenine to Threonine at nucleotide position 1762 (A1762T) and Guanosine to Adenine at nucleotide position 1764 (G1764A) [15]. These mutations were also observed in few patients with CHB and LC diagnosis as well. The presence of the above mutations in these cases can be used to identify patients more prone for development of HCC. Despite its importance in HCC development, the clinical significance of the genetic variability of the *HBV X genetic* region still remains poorly understood [59].

**Table 9:** Summary of the association of various mutations with the HBV diagnosis groups.

Gene	Amino acid substitution	AC		CHB		LC		HCC		pValue by Fisher's Exact Test	
		Frequency	Percentage	Frequency	Percentage	Frequency	Percentage	Frequency	Percentage		
**Core**	S12T	-	-	37	53.6	3	27.3	1	20	<0.05	A
**Core**	T49S	-	-	40	58	4	36.4	1	20	<0.05	A
**Core**	V105I	-	-	43	62.3	3	27.3	1	20	<0.01	A
**Core**	V116I	-	-	50	72.5	3	27.3	1	20	<0.01	A
**Core**	R172Q	-	-	16	23.2	6	54.5	1	20	<0.05	B
**Core**	Q184S	-	-	7	10.1	6	54.5	1	20	>0.05	B
**Core**	C185M	-	-	1	1.4	2	18.2	1	20	<0.05	B
**Core**	*186L	-	-	0	0	2	18.2	1	20	<0.05	B
**X**	P11S	-	-	4	5.7	3	37.5	1	20	<0.05	B
**X**	S31A	-	-	3	4.3	3	37.5	0	0	<0.01	B
**X**	G32R	-	-	5	7.1	3	37.5	1	20	<0.05	B
**X**	S33P	-	-	34	48.6	2	25	1	20	<0.05	A
**X**	T36A	-	-	12	17.1	4	50	4	80	<0.01	C
**X**	G50R	-	-	3	4.3	0	0	3	60	<0.01	C
**X**	H94Y	-	-	2	2.9	3	37.5	1	20	<0.01	B
**X**	K130N	-	-	5	7.1	1	12.5	2	40	>0.05	C
**Surface**	T118V	4	28.6	8	11.4	0	0	0	0	<0.05	D
**Surface**	V190L	3	21.4	0	0	0	0	0	0	<0.01	D
**Surface**	S204R	1	7.1	1	1.3	3	37.5	0	0	<0.01	B
**Surface**	S207N	6	42.9	18	0	1	0	0	0	>0.05	D
**Surface**	L213I	1	7.1	3	3.8	3	37.5	0	0	<0.01	B
**Polymerase**	A21S	0	0	14	17.7	3	37.5	0	0	>0.05	B
**Polymerase**	L69L_X	5	33.3	11	13.9	1	12.5	0	0	<0.05	D
**Polymerase**	T70X	5	33.3	11	13.9	1	12.5	0	0	<0.05	D
**Polymerase**	N76Q	0	0	2	2.5	2	25	0	0	<0.05	B
**Polymerase**	S81R	5	33.3	10	12.7	1	12.5	0	0	<0.05	D

NB: A- CHB Vs Others , B- LC Vs Others, C- HCC Vs Others, D- AC Vs Others.

HBV ***precore/core*** *orf* codes for HBcAg and HBeAg. HBcAg is involved in capsid formation and packaging of the pregenome reverse transcriptase complex. Precore mutants had an intermediate frequency in our study group (40%). Such mutants were found with high frequency in patients infected with genotypes D, and at a lower frequency in genotype A-infected patients. The occurrence of this mutation is dependent upon the nucleotide (cytosine or thymine) at position 1858, which forms a base pair with nt 1896 in the pregenomic RNA loop at the ε encapsidation sign. A thymine at position 1858 is particularly common in genotype D viruses. In our study we have observed the precore mutation in 46% (56/121) of HBV Genotype D. The presence of a cytosine at position 1858 precludes the G-to-A mutation at nt 1896, since this would destabilize the stem-loop structure of the RNA encapsidation signal [61]. Genotype A usually shows a cytosine at this position. Therefore, in HBV genotype A, the G1896A mutation usually arises together with a C1858T nucleotide exchange [62]. This explains the presence of precore mutation in 25% (17/66) of HBV Genotype A patients in our study group.

In another study reported from India (S Ghosh etal) [63] which selectively included 60 HBeAg –ve patients with Genotype D HBV infection, reported similar frequent mutation profile as the present study. However the present study due to inclusion of consecutive naïve chronic HBV patients could provide the differences of such mutation across the prevalent genotypes.

We also observed few de novo mutations associated with drug resistance in the patients included in the present study. All the patients were naïve and the HBV DNA was isolated from them before any therapy was instituted. Forty two patients out of the 187 in whom the mutation profile for reverse transcriptase region was analyzed had such mutations. Similar mutations have been reported earlier in several independent studies. L80* which was reported to be associated with lamivudine resistance [64] and enhanced viral replication *in vitro* was observed in 17 cases. Entecavir associated drug resistant mutations I169N, A181G, S202I reported earlier were observed in 4 cases [65]. rtN236T a primary mutation in the D domain, rtA181T/V at the B domain and rtQ215S at the C–D inter-domain have been associated with Adefovir and Lamivudine resistance were also observed in 8 cases [66], [67]. All of the above mutations were observed in patients before therapy was started. The mutations discussed above were present in treatment naïve patients. This population forms almost 22% (42/187) of the patients studied, which indicates that almost quarter of our patients included in the present study had resistance to various oral antivirals against HBV. This knowledge is also important to determine the therapy of these patients to avoid primary antiviral failure and selection of the resistant virus.

In all ten ***novel mutations*** were also identified (Table 8). The mutations in the polymerase region (D45L, T70x and S74X) were present in the amino- terminal domain of DNA polymerase that serves as a primer for Reverse Transcriptase (RT). Mutations in this region can affect viral DNA synthesis [68]. The mutations in core region(T49S and E64N) were also present in the amino-terminal domain of the core protein, which is required for the assembly and the stability of the nucleocapsid [69]. The prevalence and significance of these mutations is unclear.

A rare recombinant of HBV where the entire HBx peptide from genotype A is replaced with HBx from genotype D was also identified. Inter-genotypic recombination has been reported earlier between different groups (A/C, B/C, D/C etc) in various parts of the world [6], [70], [71], [72], [73], [74]. Such recombination events have potential of generating diversity of infecting strains thereby making diagnosis and or treatment difficult. In few studies recombinants have also been associated with development of HCC [75], [76].

## Conclusion

Among the naïve patients infected chronically with HBV, genotypes D and A were frequent in our study. The majority (55%) of the HBV-infected individuals in the present study were HBeAg-negative in all the age groups studied. The MFI approach used in the study gives an overall estimate of the conserved and variable regions in the genome. Mutations in HBV genome were more frequent in surface and polymerase genes and these frequent mutations were more often observed with advanced stages of liver disease like LC and HCC. Spontaneous drug associated mutations implicated in resistance to antiviral therapy were also identified in about quarter of our patients, which is of therapeutic concern. Few mutations in X gene (T36A and G50R) showed high frequency of association with HCC, however further studies are required to validate the findings.

The present study indicates that mutant HBV infection (HBeAg –ve; Precore/Basl Core Promoter mutants, HBsAg mutants, drug resistant mutants and mutants associated with HCC) are frequent among Indians with chronic HBV infection. Such virus may be getting transmitted to people in general and may cause diseases difficult to treat with more progressive course in future.
